# Criteria for the definition of Pituitary Tumor Centers of Excellence (PTCOE): A Pituitary Society Statement

**DOI:** 10.1007/s11102-017-0838-2

**Published:** 2017-09-07

**Authors:** Felipe F. Casanueva, Ariel L. Barkan, Michael Buchfelder, Anne Klibanski, Edward R. Laws, Jay S. Loeffler, Shlomo Melmed, Pietro Mortini, John Wass, Andrea Giustina, On behalf of The Pituitary Society, Expert Group on Pituitary Tumors, Alin Abreu Lomba, Julio Abucham, Cristina Alvarez-Escola, Ariel L Barkan, Albert Beckers, Anat Ben-Shlomo, Ignacio Bernabeu, Martin Bidlingmaier, Nienke Biermasz, Beverly Biller, Cesar Luiz Boguszewski, Marek Bolanowski, Jens Bollerslev, Vivien Bonert, Marcello Bronstein, Oscar D. Bruno, Michael Buchfelder, John D. Carmichael, Philippe Caron, Philippe Chanson, Felipe F. Casanueva, Richard N. Clayton, Annamaria Colao, Fernando Cordido, Laura De Marinis, Rudolf Fahlbusch, Maria Fleseriu, Anna Maria Formenti, Pamela U. Freda, Hidenori Fukuoka, Ezio Ghigo, Andrea Giustina, Yona Greenman, Elena Grineva, Ashley Grossman, Mark Gurnell, Anthony Heaney, Andrew R. Hoffman, Irena Ilovayskaya, Gudmundur Johannsson, Pinar Kadioglu, Niki Karavitaki, Laurence Katznelson, Fahrettin Kelestimur, Daniel F. Kelly, Anne Klibanski, Ken Ho, Michal Krsek, Andre Lacroix, Edward R. Laws, Jay StevenLoeffler, Marco Losa, Jens Otto Jørgensen, Anton Luger, Susana Mallea-Gil, Adam Mamelak, Gherardo Mazziotti, Ann McCormack, Shlomo Melmed, Moises Mercado, Pietro Mortini, Sebastian Neggers, Guang Ning, Nelson M. Oyesiku, Vera Popovic, Milan Petakov, Stephan Petersenn, Misa Pfeifer, Antonio Pico, Manuel Puig Domingo, Gérald Raverot, Martin Reincke, Monica Roberto Gadelha, Roberto Salvatori, Susan L. Samson, Akira Shimatsu, Ilan Shimon, Paul Stewart, Christian Strasburger, Brooke Swearingen, Peter Trainer, Nicholas A. Tritos, Stylianos Tsagarakis, A. J. van der Lely, Lucio Vilar, Rocio Villar-Taibo, John Wass, Maria Chiara Zatelli

**Affiliations:** 1 Division of Endocrinology, Santiago de Compostela University, Santiago de Compostela, Spain; 2 Division of Endocrinology, University of Michigan Health System, Ann Arbor, MI USA; 3 Department of Neurosurgery, University Hospital Erlangen, Erlangen, Germany; 4 Neuroendocrine Unit, Massachusetts General Hospital, Boston, MA USA; 5 Department of Neurosurgery, Brigham & Women’s Hospital, Boston, MA USA; 6 Department of Radiation Oncology, Massachusetts General Hospital, Boston, MA USA; 7 Cedars-Sinai Medical Center, Los Angeles, CA USA; 8 Department of Neurosurgery, San Raffaele University Health Institute Milan, Milan, Italy; 9 Oxford Centre for Diabetes, Endocrinology and Metabolism, Churchill Hospital, Oxford, UK; 10 Division of Endocrinology, San Raffaele University Hospital, Milan, Italy

**Keywords:** Acromegaly, Cushing’s disease, Thyrotropinomas, Gonadotropins, Transsphenoidal surgery, Pituitary radiotherapy

## Abstract

**Introduction:**

With the goal of generate uniform criteria among centers dealing with pituitary tumors and to enhance patient care, the Pituitary Society decided to generate criteria for developing Pituitary Tumors Centers of Excellence (PTCOE).

**Methods:**

To develop that task, a group of ten experts served as a Task Force and through two years of iterative work an initial draft was elaborated. This draft was discussed, modified and finally approved by the Board of Directors of the Pituitary Society. Such document was presented and debated at a specific session of the Congress of the Pituitary Society, Orlando 2017, and suggestions were incorporated. Finally the document was distributed to a large group of global experts that introduced further modifications with final endorsement.

**Results:**

After five years of iterative work a document with the ideal criteria for a PTCOE is presented.

**Conclusions:**

Acknowledging that very few centers in the world, if any, likely fulfill the requirements here presented, the document may be a tool to guide improvements of care delivery to patients with pituitary disorders. All these criteria must be accommodated to the regulations and organization of Health of a given country.

## Introduction

The pursuit of excellence is a continuous endeavour for health professionals and is increasingly sought by society and health administrators alike. It is widely accepted that only experts in a field may provide the best standard of care to patients. In this context, pituitary tumors are more frequent than previously thought [1, 2] and present a significant challenge for diagnosis and management. There is a widely held consensus endorsed by publications in the field, indicating that the best care for these patients is provided by an interdisciplinary team composed of dedicated endocrinologists and experienced pituitary surgeons working in collaboration [3, 4]. Such a core team needs to be supported by a collaborative environment of specialists in other areas, such as neuroradiology, neuropathology, radiation oncology, neuro-ophthalmology, otorhinolaryngology, plus trained nursing [4].

The goals of that team should include: (1) early detection of the tumor; (2) establishing the diagnosis; (3) determination of the most suitable treatment, which can either be observation; surgical, medical or radiotherapy; (4) if surgical, removing the pituitary mass while preserving the normal pituitary tissue and nearby structures; (5) the surgical or medical treatments working alone or concomitantly must eliminate hormonal hypersecretion and/or its effects; (6) prevention of tumor recurrence; and (7) recognizing and caring for the acute and delayed complications of the disease, especially hypopituitarism. The final goal is the elimination or at least reduction of the excess morbidity and mortality associated with the tumor and hypersecretion syndrome as well as treatment of accompanying pituitary hormone insufficiencies [3–5]. For many patients, this requires a program of care, including medical therapy, surgery and radiation therapy, in addition with long-term follow-up.

## The need for Pituitary Tumor Centers of Excellence (PTCOE)

The concept of a Center of Excellence has been promulgated in various fields of medicine to address public and professional concerns regarding the quality of care of a given group of patients. This concept has been found to be useful primarily in activities involving multidisciplinary teams and more often in teams that are composed of experts in surgical techniques and medical treatments [6–8]. In some centers treating pituitary tumors, high-level interdisciplinary groups are already developed and effectively function, but in most cases, these groups are self-appointed structures without formal acceptance by hospital managers, or health authorities, no by their colleagues, and there are no formal definitions of the criteria needed to be considered truly “excellent” [4]. If there are no explicit requirements for achieving a degree of excellence, patients’ outcomes cannot be accurately measured, and the fulfillment of the previously outlined goals cannot be determined. In addition, the evaluation and accreditation of such structures by external and independent bodies are not performed.

Despite efforts to foster improved adherence to the recommended standards being available, a major barrier to optimal care in patients with pituitary tumors is a delivery system that too often is fragmented, lacks clinical information capabilities, duplicates services, and is not well designed for the delivery of co-ordinated chronic care.

The relationship between quality and outcome has been intuitively affirmed by endocrinologists who have a “preferred pituitary neurosurgeon” to whom they refer patients. However, in a given hospital, efforts by endocrinologists or administrators for example to limit surgical procedures to a single surgeon, may encounter firm opposition by the neurosurgery staff stating that “a graduate in Neurosurgery can perform any intervention”. In such situations, surgeons perform all of the activities without sub specialization. In the absence of external guidelines generated and endorsed by authoritative bodies, the status quo is unlikely to change.

A relevant point when dealing with the concept of a PTCOE is performance of scholarly scientific discovery and the transparent communication of results and outcomes. Units formed by a lone endocrinologist or an individual neurosurgeon will have difficulty communicating results to symposia and administrative organizations. This is a crucial point in eliminating “communication bias”, i.e., the fact that small groups or groups that perform poorly never present results. Therefore, the literature is full of reports from the more successful groups in the world with the best outcomes and this bias contributes to the fact that there is limited understanding of the true results when evaluating outcomes of new drugs or new surgical approaches. In this context, the implementation and use of disease-specific registries and electronic clinical files may provide a reliable tool with which to communicate unbiased results. As part of their mission, endocrinologists, as well as neurosurgeons and other specialists caring for patients with pituitary disorders, have a duty to make progress in the pituitary sciences, and this is only attainable through teamwork.

Articles indicating the need for Centers of Excellence for pituitary diseases have mostly focused on surgical procedures outcomes [9–15]. Despite convincing arguments, such efforts have not yet been able to change the general surgical practice. On occasion, health or administrative authorities have attempted to define the characteristics of such centers of excellence, but frequently, the main goal was to reduce costs, and these characteristics have not been accepted by the professionals involved in the day-to-day care of the patients. For such reasons, a practical and effective definition of a Pituitary Tumor Center of Excellence (PTCOE) is needed, and the characteristics of such are summarized herein.

**Table:** BOX 1. General characteristics of a PTCOE are:

Provide the best care for patients with pituitary tumors and related pathologies Independent of health authorities, administrations and for-profit organizations Widely recognized by endocrinologists and pituitary surgeons Aimed to the advancement of pituitary science Providing adequate patient education and community outreach Recognized by external national and/or international endocrine and neurosurgical medical societies Act as training center for residents in the treatment of pituitary pathologies	

Based on the characteristics outlined above, we conclude that to provide the best care to patients with pituitary tumors, it is desirable to identify PTCOEs in a given health system area. For a group of endocrinologists who specialize in pituitary disorders and pituitary surgeons participating in such units, it is mandatory to know in an explicit way the requirements and conditions needed to develop such a center and to evaluate its success. These need to be established by an external learned body that may or may not perform the final step of validation of the center.

**Table:** BOX 2. Mission of the PTCOE

1. Provide the best standard of care to patients with pituitary tumors and disorders 2. Organize multidisciplinary clinical management 3. Liaision between experienced neurosurgeons and expert neuroendocrinologists 4. Work with the supporting specialties 5. Train fellows in the management of pituitary tumors and related disorders 6. Provide courses, publications and lectures for primary care physicians and other specialists 7. Capture and track clinical data 8. Provide up to date and comprehensive patient information 9. Present results and outcomes to scientific bodies and administrators 10. Support endocrine units located outside the PTCOE 11. Advise health administrators and authorities on specific problems 12. Advance the science and scholarship of pituitary tumors 13. Include tumor data on National or Regional registries	

A PTCOE need to envisioned on a “patient centric” organization, as the patient is the core of its mission Patient Networks, engagement activity, family impact, educational platforms, digital infrastructure to facilitate care across primary-secondary health care, and across specialties are essential for a properly focused PTCOE.

## Excellence in pituitary surgery

Although advances in the medical treatment of pituitary tumors over the last decades have been stellar, it is beyond doubt that a PTCOE is dependent on the presence of a dedicated and excellent group devoted to pituitary surgery by the endonasal transsphenoidal or transcranial approaches.

In fact, assuming that modern techniques of imaging, and neuronavigation are available at a given center, the presence of an experienced neurosurgeon capable of performing pituitary microsurgery in a safe and effective manner is mandatory. Pituitary surgery is the most effective procedure for acromegaly, Cushing’s disease, TSH-secreting adenomas, resistant prolactinomas and non-functioning pituitary adenomas causing mass effects. It is also effective for pituitary apoplexy, diagnostic uncertainly as to the nature of the lesion and, in the rare cases of pituitary cancer. This approach is also recommended for para-sellar pathologies, such as craniopharyngiomas, Rathke cleft cysts, some chordomas and some skull base meningiomas [9].

**Table:** BOX 3. Targets of pituitary surgery

1. Eliminate pituitary hypersecretory syndromes 2. Eliminate, reduce or control the tumor mass 3. Preserve the normal pituitary gland function 4. Preserve surrounding neural structures, including the optic apparatus, other cranial nerves and parasellar vasculature 5. Reduce or eliminate acute complications generated by the tumor 6. Reduce or eliminate the risk of tumor recurrence	

Although defining targets for pituitary surgery is relatively easy, defining excellence for an individual pituitary surgeon is considerably more difficult. An excellent neurosurgeon requires a solid knowledge of hypothalamic-pituitary organ physiology and the principles of its endocrine evaluation, plus continuous practice to maintain his/her level of surgical expertise; otherwise, the quality of the surgeon’s work may deteriorate.Therefore, an experienced high-level pituitary neurosurgeon requires solid training in basic neurosurgery and continuous practice, and the latter is based on a continuous high workload and demonstrated evidence based outcomes.

The basic knowledge of neurosurgery relies on a residency program that is a *sine qua non* for a future excellent neurosurgeon. However, in most centers in most countries, the program provides limited experience in transsphenoidal pituitary surgery. This is because most residency training centers perform a small number of transsphenoidal surgeries per year. This situation does not allow the graduate to gather enough experience to be able to practice independently immediately after completing training [9].

Conversely, specific intervention to the pituitary region through an endonasal approach requires a clear understanding of skull base anatomy and the expertise necessary to maneuver instruments and the endoscope in such a narrow surgical space [16, 17]. In addition, the residency training program for a neurosurgeon is so demanding that graduates have insufficient experience in management of pituitary patients, and insufficient interactions with neuroendocrinologists. For these reasons, a graduate who wishes to have a significant pituitary tumor practice in the future should have an additional fellowship at a high-quality center performing a large number of interventions each year [18]. With such a combination of a residency plus a fellowship, the future pituitary tumor surgeon should then have the appropriate education and training.

After the residence, depending on the training opportunities available at a given country, we would recommend either (1) completion of a formal postgraduate fellowship in pituitary surgery, (2) completion of a postgraduate fellowship in skull base or neuro-oncologic surgery at a high volume pituitary center, or (3) completion of postgraduate subspecialty training at a high volume pituitary center.

**Table:** BOX 4. Basic requirements for excellence for a given neurosurgeon using transsphenoidal procedures

1. Basic residency training in neurosurgery at an accredited center 2. Post-residency fellowship (12–15 months) in an active, high-level pituitary surgery unit, or extensive training in pituitary surgery and pituitary patient management at an established pituitary center 3. Continuous practice in a newly created or previously recognized unit with a high pituitary workload and demonstrated outcomes 4. Contribute to the advancement of Pituitary Science through publications in medical journals, chapters in books and monographics	

### Pituitary surgery. Outcomes and expertise

One of the peculiarities of surgery in general, and of pituitary surgery in particular, is that if a superbly trained surgeon does not maintain a high number of operative cases in the following years, the surgeon may lose the capability of performing at an excellent level. Expertise then requires basic training, specific training and continuous practice. For such reasons, several reports have shown that experienced surgeons have better outcomes and lower rates of complications than surgeons with less experience [9, 19–24]. In a US national survey on complications of transsphenoidal surgery conducted on a large number of active neurosurgeons, respondents were divided into three groups depending on number of pituitary surgeries performed along their life-long experience. The morbidity and mortality was lower for the surgeons with more interventions, and the rate of complications was lower. Despite that, the most experienced surgeons witnessed more severe complications in absolute terms, related to the high numbers of operative procedures, and also because they operate on the most complicated cases [24]. Additionally, in cases of surgical reoperation when the first surgery is complicated or fails, surgical experience is crucial for obtaining optimal results [25–28].

After having a neurosurgeon with the correct training during residency who afterwards performs a fellowship at a high level center, what makes the difference between experienced and less experienced surgeons? The answer is the number of procedures performed by the surgeon per year. In fact, reports from UK centers that have expertise in pituitary tumors have observed that surgeons with a high workload have better outcomes. For a hospital treating a fixed population, a smaller number of surgeons produce better outcomes, and centers with only one neurosurgeon performing pituitary surgery have better outcomes than centers with several such surgeons [19, 20, 22, 29–31].

These observations have been endorsed by groups in other countries. A high workload, i.e., a high number of procedures performed each year, provides the neurosurgeon using the transsphenoidal approach with sufficient experience with respect to patient selection, operative techniques, and better outcomes, in addition to reducing the rate and severity of complications [9, 15, 21, 22, 24, 32, 33]. Finally, evaluation of efficacy must be based on outcome data [34, 35].

**Table:** Box 5. Experienced pituitary neurosurgeon for PTCOE: rationale and definition

1. Experienced pituitary neurosurgeons have better outcomes, and reduced rates of morbidity and mortality 2. The workload of a surgeon is based on the ratio between the number of operating surgeons and the size of the population served by the center 3. For a fixed number of inhabitants covered by the center, a reduced number of transsphenoidal pituitary surgeons results in better outcomes 4. The ideal number of transsphenoidal pituitary interventions for micro and macroadenomas for an individual surgeon is debatable but should be approximately 50 per year	

Assuming that expertise or excellence comes from the workload of a well-trained surgeon, and considering that most centers serve a fixed number of inhabitants, the solution proposed by several publications would be to have a limited number of surgeons devoted to pituitary tumors at each center, with a backup for emergency cases.

A single neurosurgeon situation, however, has several drawbacks. For example, the center will remain uncovered when the surgeon is absent, the training of new fellows would be difficult, and performing clinical research and communicating results could be impeded. For such reasons, one alternative could be to concentrate several neurosurgeons (two to four) at a given center that covers the transsphenoidal surgery needs of a whole region that ideally has 2.5 to 5 million inhabitants. Such a center would receive patients who were generated locally but also referred from endocrine units located at other hospitals in the region, i.e. “regionalization” for a pituitary center [11]. The regionalized center can receive patients already diagnosed, perform the intervention, and return the patient for follow up at the hospital or physician of origin. This process may be facilitated by implementation of electronic clinical registries working within a network. As the number of patients with pituitary diseases is not large, such an organization should be satisfactory in cost-benefit terms for health administrators, even considering the travel costs of the patient to the reference center.

**Table:** Box 6. Organization of a neurosurgical center for a PTCOE

1. Expertise is based on workload, i.e., the quotient between a limited number of surgeons and a high number of patients 2. An alternative to reducing the number of surgeons performing transsphenoidal pituitary procedures is to expand the population covered 3. Having more than one neurosurgeon is convenient in terms of continuing access, training new fellows and scientific progress 4. An ideal reference center for a region may be formed by 2–4 expert neurosurgeons performing transsphenoidal operations, including macro and micro adenomas serving a population of 2.5 to 5 million inhabitants, with a proportional increase in the work load 5. Such a regionalized unit can give surgical coverage to several external endocrine centers of excellence, and can return the patient to his/her local hospital for follow-up after surgery, according to follow up protocols	

### Endocrine Units of Pituitary Tumors Centers of Excellence

A PTCOE requires that the neurosurgery group works closely with an endocrinologist on an endocrine unit or a division that has a special emphasis on pituitary diseases. Obviously, pituitary diseases are not only tumors but encompass other pituitary pathologies and include the secondary effect of tumor treatment, such as central hyposecretion of TSH or LH/FSH, GH deficits, panhypopituitarism, as well as, Sheehan syndrome, diabetes insipidus, and so on, which are all managed by the same professionals [34–38]. At the end the endocrinologists need to serve a “generalist role” for a life long term and provide an holistic management for the patient. Only a combination of expert neurosurgeons working in liaison with expert neuroendocrinologists can provide the excellence of care needed to meet the definition of a PTCOE. This team will use advanced techniques of diagnoses and treatment, produce scientific reports, and present their results to scientific bodies and administrative authorities.

The mission of the endocrine component of the PTCOE includes suspecting pituitary disease, establishing the diagnosis, determining the optional treatment plan with neurosurgeons regarding surgical intervention, providing support for peri-operative care, and providing long term follow up management. Also document and manage any endocrine deficiencies across radiation procedures.

Defining expertise for an endocrinologist appears to be easier than for a neurosurgeon considering that the requiring workloadhas not be determined. It is evident, however, that an endocrinologist wishing to participate in a PTCOE needs to have received basic training in internal medicine and endocrinology though the residency stage, and then to perform postgraduate training for at least 12 months as part of a group of experts in pituitary disorders that has international stature.

The expert endocrinologist must have a through knowledge of the laboratory techiques for hormone analysis because diagnosis is based on the accurancy of such techiques. Analytical methods are complex and a true knowledge of reagents, calibrators and degree of standarization is mandatory. An adequate understanding of modern genetic techiques is also relevant for diagnosis. An expert endocrinologist must be able to rudimentary understanding of MRI studies to being able to read/interpret and review pituitary MRI studies, understand pituitary pathology, significance of positive immunostaining in absence of clinical signs- such as silent corticotroph tumors, and the importance (and limitations) of proliferative indices in regard to tumor growth.

**Fig. 1:**
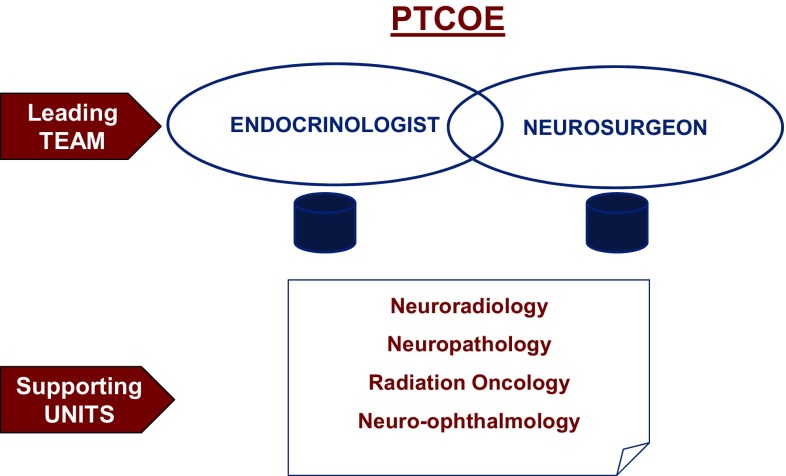
General structure of a Pituitary Tumors Center of Excellence (PTCOE). The leading team composed by the endocrinology and by neurosurgery teams, and the main supportly units of other specialities

To substantiate excellence, the endocrinologist should present results at scientific meetings and contribute to the advance of pituitary science. This can be supported by regular publications of peer-reviewed research articles, reviews, chapters in monographs or textbooks, participation in scientific or consensus meetings, as well as by regular participation in multi- center trials of novel pituitary-directed treatments strategies (Fig. 1).

**Table:** Box 7. Experience of the endocrinologist working at a PTCOE

1. Specialty medical training in internal medicine or adequate alternatives and in endocrinology 2. Postgraduate training at a center with a unit dedicated to pituitary disorders 3. Working at a PTCOE with intense activity and a high workload 4. Presenting results at scientific events and to health administration bodies 5. Contributions to the advancement of pituitary science, and discovery in pituitary medicine	

## Supporting units for a PTCOE

The close relationship of expert pituitary surgeons and pituitary endocrinologists requires the additional support of several specialties to provide a high-level standard of care. For imaging analysis, a group of well-trained neuroradiologists is ordinarily available in most centers and in all academic units. However in certain circumstances, familiarity with specific protocols to locate often small microadenomas in for example Cushing disease is of importance in a full PTCOE. For diagnosis, reports from the neuropathologist should include tumor immunocytochemistry to make full diagnosis and comments if the tumor exhibits unusually high proliferative potential as this may have bearing on subsequent management. There may be the need for close surgical collaboration between neurosurgery and otolaryngology in the execution of endonasal and other skull-base approaches. Neuro-ophthalmologists are required for the diagnosis and, in some cases, for follow-up. The contribution of such specialties is mandatory to provide a given PTCOE its necessary support [9, 39–47].

**Table:** BOX 8. Units providing support to a PTCOE

1. Neuroradiology 2. Neuropathology, including molecular diagnosis 3. Radiation neuroncology 4. Neurooncology 5. Neuro-ophthalmology 6. Specialized clinical and research nursing	

## Expert Neuroradiology Unit

At a minimum, high field magnetic resonance imaging (MRI) with at least 1.5 T field strength and high resolution depiction of the sellar region should be available for every patient with a pituitary tumor throughout the day. In addition, around the clock availability of thin-collimation computerized tomography is required for those who have contraindications to MRI. Orientation of the sections and labelling of the images should be standardized to enable reliable follow-up studies. The centre should have access to digital substraction angiograpy, and to expert selective bilateral venous sampling of the inferior petrosal sinus.

## Expert Neuropathology Unit

Tumor Pathology is an essential aspect of the diagnosis, management and follow-up of patients with pituitary and related disorders. The pathologic diagnosis guides the optimal therapeutic strategy, and helps to determine the response to treatment and the prognosis for the patient.

The ideal arrangement for Pathology to be an integral part of the multidisciplinary PTCOE would be as follows:

**Table:** BOX 9. Neuropathology unit

1. One or two experienced neuropathologists or endocrine pathologists who will have responsibility for the final diagnosis 2. Routine assessment of histology—mitoses, pleomorphism, giant cells, inclusions, inflammatory changes, stroma, hemorrhage, vascular features. Proliferative index Ki67. Routine stains 3. Routine pituitary hormone stains ACTH, prolactin, growth hormone, TSH, LH, FSH, and additionally in some cases alpha subunit, chromogranin, P53, hormone receptor stains, transcription factors 4. Tumor specimen banking 5. A standard report indicating a final diagnosis using the most current WHO guidelines and criteria with commentary on normal pituitary gland incorporated in the specimens	

## Radiation Neuroncology Units of Excellence

Radiation therapy is required to treat some pituitary tumors that are completely or partially resistant to medical treatment, or for surgical remnants from such tumors, patients who refuse or cannot undergosurgery,aggressive pituitary tumors or in pituitary cancers. In all of these cases, LINAC radiotherapy, stereotactic radiotherapy or radio-surgery, all of which are computer-assisted techniques performed by radiotherapists expert in the treatment of intracranial tumors, are necessary at a high level PTCOE [48–57].

Single fraction stererotactic radiosurgery (single dose) or fractionated stereotactic treatmentneed to be accessible from the center of excellence, to be utilized depending on patient and tumor related factors. Radiation neuroncologists treating patients with pituitary adenomas must have an in-depth knowledge of the tolerance of the optic system, cranial nerves of the cavernous sinus, temporal lobes and the normal pituitary gland. They should also understand the temporal relationship between the delivery of radiation and the interval for development of such complications as hypopituitarism. Radiation oncologists should work in close cooperation with the neuroendocrinologists and neurosurgeons.This should also allow selected radiotherapists to gain enough experience in the treatment of pituitary tumors.

In some national Health Systems, radiation neurooncology units are not located in every high level hospital but at specific centers. In such situations close relationship between radiotherapists and neuroendocrinologists must be guaranteed.

**Fig. 2:**
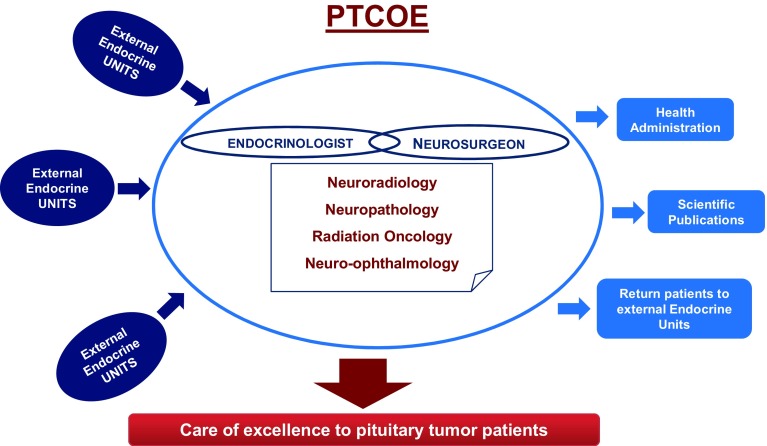
Working flow chart of a given PTCOE. The Unit of Excellence benefits of working in collaboration with external endocrine units that may be located in other centers or hospitals. After surgery patients return to their original units. This allows for a networking method of patient care

All of these different specialties working in a team with neurosurgeons and expert neuroendocrinologists form the core of a PTCOE. The internal architecture of the unit and facilities should allow intense collaborative and interdisciplinary functioning. In some national Health Systems there are regional endocrine centers of high quality that may interact with a PTCOE. A bidirectional flux the patients would guarantee the best care for patients suffering of pituitary pathologies (Fig. 2).

## Conclusions

In the last few decades, a considerable body of evidence supports the concept that patients with pituitary tumors would receive the best care from units of excellence composed of expert neurosurgeons performing pituitary surgery by transsphenoidal and other approaches, plus experienced neuroendocrinologists devoted to these types of tumors. These experts, working in liaison with supporting units, would form a center of excellence for pituitary tumors (PTCOE). Such a center would be the optimal organization for patients, the most cost-effective for health administrators, and a more suitable structure to allow for derivation and presentation of results, and advancement of pituitary science. The present document is intended to provide the basis for such a PTCOE structure.
